# Management of COVID-19 vaccines cold chain logistics: a scoping review

**DOI:** 10.1186/s40545-022-00411-5

**Published:** 2022-03-02

**Authors:** Mathumalar Loganathan Fahrni, Intan An-Nisaa’ Ismail, Dalia Mohammed Refi, Ahmad Almeman, Norliana Che Yaakob, Kamaliah Md Saman, Nur Farhani Mansor, Noorasmah Noordin, Zaheer-Ud-Din Babar

**Affiliations:** 1 Faculty of Pharmacy, Universiti Teknologi MARA, Selangor Branch Puncak Alam Campus, 42300 Bandar Puncak Alam, Malaysia; 2 Collaborative Drug Discovery Research (CDDR) Group, Communities of Research (Pharmaceutical and Life Sciences), Universiti Teknologi MARA (UiTM), Selangor Darul Ehsan, Malaysia; 3 Pharmacy Department, Prince Sultan Armed Forces Hospital, Al-Madinah Al-Munawarah, Saudi Arabia; 4 College of Medicine, Pharmacology Department, Qassim University, Buraydah, Saudi Arabia; 5 Faculty of Pharmacy, Universiti Sultan Zainal Abidin (UniSZA) Kampus Besut, 22200 Besut Terengganu, Malaysia; 6 Rhazes Consultancy Services Sdn Bhd, Seksyen U19, 40160 Shah Alam, Selangor Malaysia; 7 University of Huddersfield, Huddersfield, HD1 3DH West Yorkshire UK

**Keywords:** Cold chain logistics, mRNA vaccines, Adenovirus vector, Supply and distribution chain, Equitable access, Temperature control, Biologics

## Abstract

**Background:**

Successful mass vaccination programmes are public health achievements of the contemporary world. While pharmaceutical companies are actively developing new vaccines, and demonstrating results of effectiveness and safety profiles, concerns on COVID-19 vaccine management are under-reported. We aimed to synthesise the evidence for efficient cold chain management of COVID vaccines.

**Methods:**

The scoping review’s conduct and reporting were based on the PRISMA–ScR 2018 checklist. We searched from April 2020 to January 2022 for publications in PubMed (LitCovid), Scopus and ScienceDirect. All review stages were pilot-tested to calibrate 2 reviewers. Articles on cold chain logistics and management were included, while publications solely describing COVID vaccines, their development and clinical aspects of the vaccine, were excluded. To capture relevant data, charting was conducted by one reviewer and verified by another. Results were analysed thematically and summarised descriptively in a table and in-text.

**Results and discussion:**

We assessed 6984 potentially relevant citations. We included 14 publications originating from USA (*n* = 6), India (*n* = 2), Finland, Spain, Bangladesh, Netherlands, Switzerland and Ethiopia. They were reported as reviews (4), policy or guidance documents (3), experimental studies (2), case reports (2), expert commentary (1), phenomenological study (1), and decision-making trial and evaluation laboratory trial (1). The findings were presented in three themes: (i) regulatory requirements for cold-chain logistics, (ii) packaging and storage, and (iii) transportation and distribution. A conceptual framework emerged linking regulatory requirements, optimal logistics operation and formulation stability as the key to efficient cold chain management. Recommendations were made for improving formulation stability, end-product storage conditions, and incorporating monitoring technologies.

**Conclusion:**

COVID-19 vaccines require special end-to-end supply cold chain requirements, from manufacture, and transportation to warehouses and healthcare facilities. To sustain production, minimise wastage, and for vaccines to reach target populations, an efficient and resilient vaccine supply chain which is assisted by temperature monitoring technologies is imperative.

**Supplementary Information:**

The online version contains supplementary material available at 10.1186/s40545-022-00411-5.

## Background

The COVID-19 outbreak had single-handedly crippled healthcare systems, induced political instability, and transformed cultural and social norms worldwide. At the financial forefronts, and as part of Operation Warp Speed, the US government had invested an initial $6.5 billion (£4.8 billion equivalent) in COVID-19 therapeutics and vaccines—an effort aimed at delivering 300 million doses of vaccines, with first doses made available as early as January 2021 [1]. The amount included at least $1 billion (£738 million equivalent) each to Novavax, the University of Oxford and AstraZeneca, GlaxoSmithKline and Sanofi, and Johnson & Johnson. Similarly, the UK government in the early stages purchased a total of 340 million doses at fixed prices, while Australia had invested approximately A$3.3 billion (£1.7 billion equivalent) for five different vaccines [1].

Recently, the World Health Organisation (WHO), by observations of superspreading events, uncovered that COVID-19 transmissions are airborne and that containing the outbreak will, therefore, be challenging [2]. The organisation recommends that general ventilation is supplemented with airborne infection controls, such as local exhaust, high efficiency air filtration, and germicidal ultraviolet lights, particularly in hospitals, schools, public buildings, workplace environments, and aged care homes. On the parallel, maintaining hygiene and good hand-washing techniques, practising social distancing, efficient contact tracing and mass-testing, and droplet precautions are still advocated, at the time of writing.

Evidently, administering vaccines including booster shots on this scale and at this speed has never been done. Pharmaceutical companies are focused on meeting the demands for COVID-19 vaccine supply, and on demonstrating results of good immune responses and reasonable safety profiles of their respective candidate vaccines in all special interest groups. Up until May 2021, of the 170 vaccine candidates for COVID-19 developed, researchers were testing 90 vaccines in ongoing human clinical trials, with 27 having reached phase 3 and approved to be used for the population in various countries and currently undergoing post-marketing surveillance for adverse effect following immunisations [3]. While we constantly face the risks of adverse effects developing post-immunisation, and more potent variants emerging and creating new waves of infections, we continue to rely on the active acquired immunity offered by the COVID-19 vaccines.

The global data for COVID-19 vaccination policy demonstrated that the delivery and outreach of the vaccines for the different groups have been largely universal or that at least the vulnerable groups were targeted [4]. An exception existed for Eritrea, where none of its population was provided with the vaccine. In Afghanistan and Liberia, the vaccines were available for either their key workers or clinically vulnerable groups or the older-age groups. The Saudi government recorded that by early February 2022, 59 million doses were successfully administered, of which 1 million doses were allotted for older adults [4]. While a few countries had failed to report their data on vaccination provision, a majority of the countries had setup special task forces for managing the vaccines and related data. The scenario is no different in Southeast Asia. In Malaysia for instance, a special committee, known as the Special Committee for Ensuring Access to COVID-19 Vaccine Supply, JKVAV, is responsible for ensuring timely access to the COVID-19 vaccine supply. Alongside the JKJAV, the NPRA plays a vital role in ensuring vaccines' efficacy, quality, and safety. Six of the COVID-19 vaccines, from Pfizer-BioNTech (Cominarty), Oxford-Astrazeneca (Vaxzevria), Sinovac (CoronaVac), COVILO (Sinopharm), Johnson & Johnson (Janssen COVID-19 Vaccine), and CanSino (Convidecia) had received conditional approval from the regulatory agency [5]. Following waves of rising infections, as of 10^th^ February 2022, 98% of the country’s adult population eligible for vaccination had completed the required doses and were successfully inoculated, while 53.9% had received their booster shots [6]. The plan to vaccinate adolescents and toddlers as young as 5, and other special interest population groups has been approved and being rolled out in phases [7].

Issues related to mass production, storage and distribution of COVID-19 vaccines are, however, aspects which are under-researched and under-reported [8]. These challenges, if not addressed timely, will severely impact our race to achieve herd immunity. The "cold-chain" acts to preserve biological product quality from the time of manufacture until the point of administration by ensuring that the vaccines are stored and transported within the recommended temperature ranges. Vaccines can be categorised by the method by which they were developed, i.e., the different approaches used, such as (1) genetic vaccines—using mRNA to cause the body to produce viral proteins; (2) viral vector vaccines—using genetically modified viruses such as adenovirus to carry sections of coronavirus genetic material; (3) protein vaccines—delivering viral proteins (but not genetic material) to induce an immune response; (4) whole vaccine—through inactivated or attenuated coronavirus; and (5) repurposing existing vaccines, e.g., BCG [9]. The processes leading to a successful development and administration of the delicate and fragile vaccines are to be conducted meticulously and with care, as it involves extremes of temperature. Hence, this makes the management of its cold chain a subject matter of high priority, and the vaccines are to be handled with caution, failing which, the consequences can be detrimental to public health. The aim of this scoping review is to summarise and synthesise the evidence for efficient cold chain management of COVID-19 vaccines, and to propose relevant practical recommendations.

## Methods

The review was both conducted and reported according to the PRISMA–ScR guidance checklist (Additional file 1: Appendix S1). A review protocol was not registered considering the short time frame within which the data were retrieved. All review stages were pilot-tested to calibrate reviewers.

### Search strategy

Two reviewers IAI and MLF independently conducted the search using the three electronic databases: LitCovid (PubMed), Scopus, and ScienceDirect, with the aim of retrieving publications related to cold chain management of COVID-19 vaccines. The search was performed using a combination of keywords, MeSH terms and free texts, for example, "COVID-19 vaccine" AND “supply-chain” AND "refrigeration OR temperature-controlled supply chain OR chiller OR cold chain”. The retrieved citations were exported into Endnote version 20. The full PubMed search strategy is available in Additional file 2: Appendix S2.

### Selection process

Two reviewers (MLF and IAI) independently juxtaposed the publications retrieved against the inclusion and exclusion criteria. The inclusion criteria were (i) publications in English and published between April 2020 and January 2022; (ii) publications which focused on cold chain operations throughout the vaccine supply chain; and (iii) publications with accessible full texts. Exclusion criteria were (i) non-English language documents, (ii) publications which focused solely on COVID-19 vaccines and their development, and (iii) publications discussing the clinical aspects of the COVID-19 vaccine, which were not related to vaccine stability or did not highlight cold chain management. The reviewers were trained and a pilot with 5 papers was performed to guarantee an inter-reviewer agreement (until a Kappa score of > 0.75 was attained). MLF and IAI independently performed the screening of titles and abstracts and any discrepancies were resolved via discussion or a third reviewer consulted (KMS).

### Data collection and analysis

Data from each study was charted individually by IAI using a standardised and pretested Word data collection form and MLF verified the data (Additional file 3: Appendix S3).

Results were analysed thematically and summarised descriptively in a table and in-text.

## Results

### Search results

**Fig. 1:**
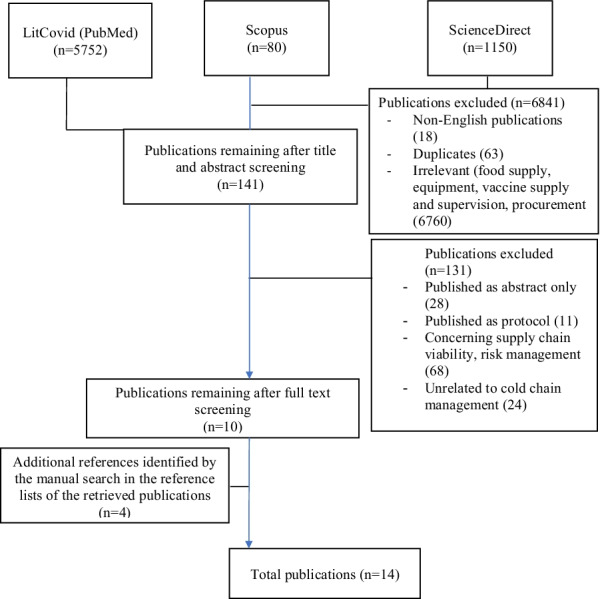
Flow chart of the literature selection process

A total of 6984 publications were retrieved from the three databases searched. After removal of duplicates, non-English texts, and screening of titles and abstracts, the number remaining were one hundred-forty-one (141) publications. Full texts remaining after review was 10. Four full-text publications were retrieved from the reference lists of relevant publications, resulting in 14 publications included this review. The flow chart for selection and elimination of publications is shown in Fig. 1. Findings from the publications were synthesised and presented in 3 themes: (i) requirements for cold-chain logistics, (ii) packing and storage of COVID-19 vaccines, and finally (iii) transportation and distribution. A conceptual framework emerged linking regulatory requirements, optimal logistics operation and formulation stability as the key to effective cold chain management (Table 1—Challenges, C1–12).

**Table 1:** Management of COVID-19 vaccines cold chain logistics

Theme/main category	Challenges (C), Authors (reference)	Origin, Article type,	Conceptual framework	Key points on challenges to cold chain management
Requirements for Cold Chain Logistics	C1 Holm MR, Poland GA. Critical aspects of packaging, storage, preparation, and administration of mRNA and adenovirus-vectored COVID-19 vaccines for optimal efficacy. Vaccine. 2021;39(3):457–9	USA, Review	Infrastructure requirements • Lack of proper storage systems	i) Each vaccine has different packaging and requirements for storage, preparation, and administration ii) Several vaccines require ultra-cold or have cold-storage requirements that are different than what vaccine administrators are prepared for
	C2 Grau S, Ferrández O, Martín-García E, Maldonado R. Accidental Interruption of the Cold Chain for the Preservation of the Moderna COVID-19 Vaccine. Vaccines (Basel). 2021;9(5):512	Spain, Case study	Formulation stability	i) No information is available on the impact on stability when vaccine is exposed to unexpected changes in temperature ii) Findings: •Accidental interruption of the storage temperature conditions had no consequences for the integrity of the mRNA contained in the Moderna COVID-19 vaccines
	C3 Yu YB, Briggs KT, Taraban MB, Brinson RG, Marino JP. Grand Challenges in Pharmaceutical Research Series: Ridding the Cold Chain for Biologics. Pharmaceutical Research. 2021;38(1):3–7	USA, Review	Temperature monitoring •Difficulties in monitoring and controlling vaccine temperature	i) Cold chain requires building extensive infrastructure and is very expensive to maintain. The complexity of the cold chain is illustrated in documents, such as the CDC Vaccine Storage and Handling Toolkit and the WHO pamphlet “The vaccine cold chain” ii) Cold chain can be standard (2 °C to 8 °C) or deepfreeze (as cold as -70 °C) iii) Cold chain breaches, if unnoticed before injection, could cause adverse drug events in patients
	C4 Rashid M. Identify constraints of vaccine supply chain: A Case study of Finnish Red Cross. 2020	Finland, Case study using qualitative method	Operational Constraints • Difficulties in monitoring and controlling vaccine temperature • Lack of proper storage systems • Inappropriate coordination with local organisations • Lack of vaccine monitoring bodies	i) Study conducted to investigate the constraints of vaccine supply chain for the Finnish Red Cross humanitarian Organization ii) Data was collected via content analysis of webpages, observation, and semi-structured interview iii) Findings: •Humanitarian organisations need to review their vaccine supply chain •IoT technology needed to improve the visibility of data flow •Real-time data and continuous monitoring of vaccine temperature is essential for developing performance of vaccine supply chain in humanitarian organisations •Cold chain equipments such as long lasting passive cold boxes, medical refrigerators, and refrigerated trucks are required for effective storage and transportation system
	C5 Crommelin DJ, Anchordoquy TJ, Volkin DB, Jiskoot W, Mastrobattista E. Addressing the cold reality of mRNA vaccine stability. Journal of Pharmaceutical Sciences. 2021 Mar 1;110[3]:997–1001	Netherlands, Commentary (Expert opinion)	Regulatory requirements	i) For vaccine licensure, the stability and expiry date of the vaccine in its final container, when maintained at the recommended storage temperature, should be demonstrated using final containers from at least three final lots made from different vaccine bulks ii) Highlights the assay capabilities and acceptance criteria for the relevant quality attributes for the proposed storage conditions for mRNA vaccines iii) In the context of the extended controlled temperature chain (ECTC) initiative of the WHO an mRNA vaccine is potentially allowed to be kept at temperatures outside of the frozen cold chain (e.g., 2–8 °C for a limited period of time) under monitored and controlled conditions
Packing and Storage	C6 Ramakanth D, Singh S, Maji PK, Lee YS, Gaikwad KK. Advanced packaging for distribution and storage of COVID-19 vaccines: a review. Environ Chem Lett. 2021:1–1	India, Review	Regulatory requirements	i) Packaging plays a crucial role in protecting, preserving, transporting, and distributing vaccines ii) Review illustrated that typical packaging and distribution system for the COVID-19 vaccine consists of different packaging levels maintaining cold storage throughout the supply chain iii) The United States Food and Drug Administration issued guidelines for vaccine manufacturers regarding storage and temperature monitoring. The end consumer should be aware of the history of the product storage conditions
	C7 Wolicki J. Vaccine storage and handling; Vaccine administration–2020. Centers for Disease Control and Prevention. 2020	US, Policy and guidance document	Operational constraints	i) COVID-19 Vaccine Addendum provides information, recommendations, and resources on storage and handling best practices to help safeguard the COVID-19 vaccine supply and ensure patients receive safe and effective vaccines ii) Vaccine storage units consist of purpose-built or pharmaceutical-grade (large or compact) •Household-grade refrigerator or freezer •Use of equipment, e.g., temperature monitoring devices presented with certificate of calibration •Handling a temperature excursion in the vaccine storage unit •Transport system recommendations •Alternatives during power outage
	C8 Training for mid-level managers (MLM). Module 1: cold chain, vaccines and safe-injection equipment management. Geneva: World Health Organization; 2008, republished 2020 under the licence: CC BY-NC-SA 3.0 IGO	Switzerland, Policy and guidance document	Operational constraints •Lack of trained personnel •Difficulties in monitoring and controlling vaccine temperature •Lack of proper storage systems	i) Highlighted the role of managers to receive vaccines and maintenance of relevant cold chain equipments. This includes for syringes, reconstitution syringes, safety boxes and vaccine diluent (which does not need to be refrigerated) ii) He or she is also responsible to adapt to changing cold storage needs and make space should a need arise
Transportation and Distribution	C9 Mohammed SA, Workneh BD. Vaccine Cold Chain Management in Public Health Facilities of Oromia Special Zone, Amhara Region, Ethiopia: Mixed Study. Journal of Drug and Alcohol Research. 2021;10[8]:1–9	Ethiopia, Phenomenological study	Operational constraints •Difficulties in monitoring and controlling vaccine temperature •Lack of proper storage systems •Inappropriate coordination with local organizations •Lack of vaccine monitoring bodies	i) 48% health facilities had satisfactory cold chain infrastructure, while 63% had good cold chain practices ii) Placement of vaccines during immunisation, checking the signs of damage, storage of food or any drugs other than vaccines, checking, availability of deep freezer, cold box and functional thermometer has a significant association with vaccine storage iii) Key informants stated that cold chain infrastructure, temperature monitoring, stock management and immunisation practice affect vaccine storage
	C10 Termini RB. An Essay on Public Health and Liberty-The Impact of the 1905 United States Supreme Court Decision of Jacobson: Compulsory Vaccination under State Police Power vs. the Individual Right to Refuse a Vaccination in this Modern-Day Era of the COVID-19 Pandemic. Widener Law Review, Forthcoming. 2021	US, Policy and guidance document	Regulatory requirements	i) Vaccine manufacturers will have to comply with the Food and Drug Authority and Federal Food, Drug, and Cosmetic Act policies ii) As the vaccines were authorised for use in an event of a public health emergency, the Emergency Use Authorization (EUA) authority will need to issue a letter of authorisation that entails two fact sheets prior to the vaccine being transported to other countries •Healthcare Providers Administering Vaccine (Vaccination Providers) •Recipients & Caregivers
	C11 Alam ST, Ahmed S, Ali SM, Sarker S, Kabir G. Challenges to COVID-19 vaccine supply chain: Implications for sustainable development goals. International Journal of Production Economics. 2021 Sep 1;239:108,193	Bangladesh, quantitative research using decision-making trial and evaluation laboratory (DEMATEL) method	Operational constraints • Difficulties in monitoring and controlling vaccine temperature • Lack of proper storage systems	i) Inappropriate coordination with local organisations, lack of vaccine monitoring bodies, difficulties in monitoring and controlling vaccine temperature, and vaccination cost and lack of financial support for vaccine purchases are the most critical challenges
	C12 Dadari IK, Zgibor JC. How the use of vaccines outside the cold chain or in controlled temperature chain contributes to improving immunization coverage in low- and middle-income countries (LMICs): A scoping review of the literature. Journal of global health. 2021;11	US, scoping review	Temperature monitoring	i) Review synthesised the evidence on how the use of vaccines outside the cold chain or in a controlled temperature chain increases immunization coverage in low- and middle-income countries (LMICs), with a focus on the timelines of the Global Vaccine Action Plan (2011–2020) ii) Vaccines can be used in: (a) controlled temperature chain (CTC) or (b) outside the cold chain (OCC)
	C13 Sun J, Zhang M, Gehl A, Fricke B, Nawaz K, Gluesenkamp K, et al. COVID-19 vaccine distribution solution to the last mile challenge: Experimental and simulation studies of ultra-low temperature refrigeration system. International Journal of Refrigeration. 2022;133:313–25	US, experimental study	To overcome operational constraints •Experimented on commercially available products, such as refrigeration container units, and retrofitted them to meet the vaccine storage temperature requirement •Simulation studies conducted, where a testing platform was setup to assess the technical merits of the solution with the ability to control temperature at − 30 °C or − 70 °C as part of the last mile supply chain	i) Refrigeration storage container test unit was fully instrumented with thermocouples, oxygen and carbon dioxide (O_2_ and CO_2)_ sensors inside the container box, scales to measure dry ice weights during sublimation and Omega 5 V differential pressure monitor to measure the pressure difference from inside and outside the container ii) O_2_ and CO_2_ sensors located near front at reefer were used in testing with a 5-psi diaphragm compressor to pump air from inside the container to outside iii) Lab tests were conducted to evaluate the performance of the proposed refrigeration storage container method. Aspects of temperature distribution of test unit, temperature distribution inside the vaccine dry-ice package, dry-ice sublimation, and CO_2_ concentration inside the refrigerated storage container were evaluated iv) Findings: •An effective, secure, and safe ultralow-temperature refrigeration solution that uses commercially available refrigeration container units used in the global cold-food chain can be modified to meet the requirements for vaccine storage to support the vaccine distribution supply chain
	C14 Nadimuthu LPR, Victor K. Environmental friendly micro cold storage for last-mile COVID-19 vaccine logistics. Environmental Science and Pollution Research. 2021	India, experimental study	To overcome operational constraints •A novel design, development, and experimental investigation of solar photovoltaic powered thermoelectric-based micro cold storage as a COVID-19 vaccine carrier for rural areas	i) An environmentally friendly, solar photovoltaic powered thermoelectric-based micro cold storage, which can function as a COVID-19 vaccine carrier for rural areas demonstrated that last-mile vaccine delivery was successfully done without any vaccine degradation

A descriptive summary was organised by aspects of: (i) cold chain requirements (process flow, regulatory requirement, logistics), (ii) packing and storage (specific for four different vaccines), and (iii) transportation and distribution (operational and logistics).

### Requirements for cold chain logistics

In this section, the regulatory and operational requirements for logistics are described.

Vaccines need to be administered by healthcare providers in clinics or pharmacies, while many other biologics for treating chronic conditions, such as diabetes and arthritis, were frequently delivered for self-injection at the patient’s home. When arriving at healthcare facilities, COVID-19 vaccine immunogenicity and effectiveness are highly dependent on the following factors: vaccines must have been stored in the required cold chain, the cold chain must be adequately monitored, and vaccines must be used up within critical timeframes after being removed from the cold chain or after a puncture in the multidose vial [9, 10]. By having appropriately trained managers on cold chain equipment who are tasked to manage and monitor cold chain, accidental interruption of the storage temperature conditions and hence vaccine instability can be avoided. Consequently, fewer vaccines are rendered ineffective and wasted. [10, 11].

Cold chain can be standard (2 °C to 8 °C) or deepfreeze (as cold as −70 °C). Cold chain requires building extensive infrastructure and is very expensive to maintain. The complexity of the cold chain is illustrated in documents, such as the CDC Vaccine Storage and Handling Toolkit [12]. Effective management of cold chain logistics for vaccines will require precise coordination across processes to ensure that the efficacy of the vaccines is preserved through temperature-monitoring and up-to-date records for traceability [7]. Constraints currently exist in their production, multimodal transport, storage, distribution and equal disbursement of vaccines to those needing them. In the European Commission (EC)-funded CHILL-ON project, most of the deviations from high-quality standards of perishable goods (vaccines included) occurred during the shipment and transportation process [13]. Where a deviation was detected, it was attributed to a lack of appropriate cold chain equipment, such as cold chain boxes, cold chain trucks and efficient refrigeration system [14]. In addition, the role of managers who receive vaccines and maintain the relevant cold chain equipment deserves recognition. They are also responsible for the reconstitution syringes, safety boxes and vaccine diluents (which does not need to be refrigerated) delivered. In addition, the manager is tasked to addressing changing cold storage needs and making additional space should a need arise [11].

Ideally, supply-chain managers will have obtained advance approvals for acquisition of these medical countermeasures—the COVID-19 vaccines. In preparedness for subsequent distribution, transport corridors which function as vital routes to end-users are identified; primary and secondary transport modes from point of entry to points of distribution are evaluated. In doing so, assessments of the respective governments’ and non-governmental organisations’ capacity to respond to emergencies, including inspection policies, customs, port, air, rail and road operations, storage, vaccine supply, communications, electricity and fuel generation, supply and distribution will also be weighed in [15]. Understanding each systemic link in the supply chain, including their strengths and shortfalls, with potential solutions or alternatives are essential. In addition, the functional linkages between governments, UN agencies, NGOs, and private sector entities are all crucial aspects of operations. It is also crucial to understand the extent of business continuity efforts at both regional and national levels at the geographical location of distribution [13, 16].

### Packing and storage of COVID-19 vaccines

Here, we present examples of four of the most common vaccines that have special packaging requirements suitable for storage and transport throughout the cold chain, from manufacturer to shipping to warehousing. Primary packaging materials include glass vials and syringes, along with stoppers and seals. Packaging for distribution, includes secondary and tertiary packaging for vaccines. Secondary packaging assist in reducing volume, cost-saving, minimising logistical burden, and reducing carbon footprint. Vaccine storage units at the healthcare facilities site usually consist of purpose-built or pharmaceutical-grade (large or compact) or household-grade refrigerator or freezer. [17].

#### Pfizer’s mRNA vaccine

Pfizer’s mRNA vaccine demands the most stringent storage needs. It is required to be stored in a − 70 °C ultra-cold freezer. Packed as a 2 mL, glass preservative-free vial containing 5 doses, the Pfizer mRNA vaccine are packed for delivery in trays of 195 vials each. Five trays of 4,875 doses will be included in each shipment of dimensions 15.7″ L × 15.7″ W × 22″ H and will require to be packed with dry ice and weigh approximately 34 kg. The vials are subjected to quality measures so no broken vials are present. Without opening the outer packaging (with the exception of inspecting the vial once to see if any is broken), each shipper can be stored for up to 10 days. Dry ice are replenished if the shipper is stored in a warmer climate and/or is opened more frequently than once for inspection of vials. GPS-enabled temperature-monitoring devices are placed inside to ensure end-to-end distribution occurs within the required temperature range. Upon arrival of the shipper, the vaccine must be transported into an ultra-cold freezer within 5 min. Simultaneously, the GPS-enabled logger is disabled and the shipper sent back to the supplier within 10–20 days of arrival. The vaccines can be thawed in the refrigerator (2–8 °C) for up to 5 days (120 h), after which it should be discarded. Each dose needs to be diluted with normal saline before use and is stable for up to 6 h at room temperature, after which time it should be discarded. Pfizer has incorporated a QR code linked to the Emergency Use Authorisation (EUA) website, a lot number, and an expiration date on the label for each vial for documentation purposes [9, 18]. Pfizer-BioNTech recently submitted data to the Food and Drug Administration (FDA) to update the storage requirements to a more reasonable temperature ranging anywhere between − 25 and − 15 °C [19].

#### Moderna’s mRNA vaccine

Packed as 10 vials of 10 doses in each carton, the Moderna’s mRNA vaccine will be shipped and delivered at a temperature of − 20 °C. One hundred doses are loaded in a carton of dimensions 5.5″ L × 2.2″ W × 2.5″ H. One advantage these vaccines have is that there are no special requirements for reconstitution or preparation and the vaccines can be stored for up to 30 days in the refrigerator at 2–8 °C until ready for use. At room temperature post-thaw, the vaccine is stable for up to 12 h, after which it should be discarded. The vial will have a QR code printed on the label. When the QR code is scanned with a smart device (i.e., phone or tablet), it will link the device to the EUA-specified website. The website will contain product information and provide access to the fact sheets. In addition, since the expiration date will not be printed on the vial, there will be a function to search for the date on the website by entering the product lot number. The carton in which the vaccines were shipped will display the same QR code as the label. In addition, the carton will have a 2D barcode printed, which is encoded with the GTIN (product ID), lot number, and an expiration date that is hard-coded to 12/31/2069. [9] Moderna has initiated a trial with a vaccine that may be refrigerator-stable. [20].

#### Johnson & Johnson’s adenovirus-vectored vaccine

Packed as 10 vials per carton and 48 cartons per shipper case of dimensions 3.66″ L × 1.5″ W × 2.13″D, the Johnson & Johnson’s adenovirus-vectored vaccine will be delivered at a temperature of − 20 °C. Each shipper will contain 2,400 doses with five doses in each vial. The J&J vaccine require to be transported to the refrigerator upon arrival. It can be stored for up to 3 months in the refrigerator (2–8 °C). Stability information at room temperature is still forthcoming. [9].

#### AstraZeneca (AZ)’s adenovirus-vectored vaccine

AstraZeneca (AZ)’s adenovirus-vectored vaccine will be shipped in pallets. Each pallet will contain 85 cases packed with 20,400 vials. 10 vials per carton will contain 100 doses. Case dimensions are 11.6″ L × 9.3″ W × 7.4″ H. AZ’s vaccine is required to be stored in the refrigerator at 2–8 °C upon arrival. It should be light-protected and can be stored for up to 6 months in the refrigerator (2–8 °C). To prevent prolonged light exposure, the vaccine must be kept in the original packaging until use and is not to be frozen. No reconstitution or special preparation is required. After the vial is punctured, it can be stored in the refrigerator for up to 6 h, after which the vaccine must be discarded. One advantage is that no reconstitution or special preparation is required for this formulation. [9].

### Transportation and distribution

In the following section, we present vaccine regulatory and logistic requirements for transportation by air, ocean and ground networks in a pharmaceutical-graded, temperature-controlled supply chain to healthcare facilities and other points of administration.

First and foremost, the vaccine manufacturers will have to comply with the Food and Drug Authority and Federal Food, Drug, and Cosmetic Act policies. As the vaccines were authorised for use in an event of a public health emergency, the Emergency Use Authorization (EUA) authority will then issue a letter of authorisation that entails two fact sheets prior to the vaccine being transported to other countries, namely:

(i) Healthcare Providers Administering Vaccine (Vaccination Providers)
(ii) Recipients & Caregivers [21]

There exists multiple stakeholders in the cold chain transportation processes, namely, the sender, freight forwarder, air and road cargo transportation companies, as well as the receiving ground handling agents [22]. They operate optimally within a risk management taskforce which is responsible for environmental health and safety, public and government affairs and policies, communications, and crucially business continuity, operations and supply chain management. Each stakeholder has a very specific role to play to complete the entire process flow. There is a risk that the operation ceases, should any of the processes fail to run its course, which then jeopardises the business entity. Exposed biological products such as vaccines and equally other biological goods to unfavourable temperatures can damage the products and eventually render them being of no use [23].

The International Air Transport Association (IATA) and its members are responsible for transporting the vaccine consignments from the nation supplying to the destination airport. Several personnel are trained on the technicalities and assist with electricity supply to the refrigerators throughout the flight. Once landed, a local road transportation company will provide refrigerated trucks to transport vaccines to and from the warehouses and the airport [24]. The trucker and assistants are then responsible to maintain vaccines within recommended temperature ranges. Local government officials provide on-field assistance for customs clearance of vaccines at the airport, and this sometime involves humanitarian organisations which are responsible for distribution to those who need them [25]. At the healthcare facilities, the capacity of cold chain equipment will also need to be upgraded; to cite an example, in Ethiopia, only 48% health facilities had satisfactory cold chain infrastructure, while 63% had good cold chain practices [26]. In addition, inappropriate coordination with local organisations, lack of vaccine monitoring bodies, difficulties in monitoring and controlling vaccine temperature, and financial support for vaccine purchase were identified as the main challenges in the region of Asia [27]. Another area which is also under-researched is the use of vaccines in controlled temperature chain (CTC) or outside the cold chain (OCC) environments [23]. One study experimented on commercially available products, such as refrigeration container units, and retrofitted the test units to meet the vaccine storage temperature requirement. Experimental and simulation studies were conducted to assess the technical merits of the solution with the ability to control temperature at − 30 °C or − 70 °C as part of the last mile supply chain [28]. In India, an environmentally friendly, solar photovoltaic powered thermoelectric-based micro cold storage which can function as a COVID-19 vaccine carrier for rural areas, was designed and an experimental study conducted. From the study, last-mile vaccine delivery was successfully done without any vaccine degradation [29].

## Discussion

Notably, all the included publications in our scoping review were available in LitCovid, an open database of COVID-19 literature, which is essentially a curated literature hub, to track up-to-date scientific information in PubMed. We identified 14 publications originating from USA (6), India (2), Finland, Spain, Bangladesh, Netherlands, Switzerland and Ethiopia. They were reported as reviews (4), policy or guidance documents (3), experimental studies (2), case reports (2), expert commentary (1), phenomenological study (1) and decision-making trial and evaluation laboratory trial (1).

## Recommendations for best practice

### Equitable access and public health policy

There are justifiable concerns that the requirements for recipient countries to maintain deep-freeze production, storage and transportation networks, in particular for the Pfizer vaccines, will limit the capacity of distributors from transporting the vaccine to low- and middle-income countries. The success in distributing vaccines to remote areas lies in the mechanisms in place to prevent unavoidable exposure to many stresses such as temperature, light, and agitation that may result in loss of vaccine effectiveness. Warm climate regions and poor intercity-connectivity pose obstacles to delivering the temperature-sensitive biologics to the public. In Peru, for example, 30 ultracold freezers existed, but none outside of Lima city. These specialised freezers will take 4–6 weeks to produce and cost between $10,000 and $25,000. One of the proposed solutions is, therefore, to top off containers with dry ice every 5 days to keep temperatures stable although this is not a practical solution as dry ice may be scarce in rural areas, and shipping dry ice, which sublimates and transforms into carbon dioxide gas, is costly and dangerous [30]. Second, since the infrastructure for 2–8 °C is already in place in many communities worldwide, as opposed to ultracold logistics, low-to-middle-income countries may opt for COVID-19 vaccine candidates requiring the minimal cold chain requirement. Innovation was key—in India, for example, companies that dealt with food chain and had cold storage facilities were compelled to innovate and were subjected to a government directive to do a minor redesign of storage spaces to accommodate such need [31]. Third, advanced cold chain equipment such as long lasting passive cold box, medical refrigerators, and refrigerated trucks can facilitate effective storage and transportation system.

### Technologies to assist cold chain logistics

Practising data logging does ease the process of monitoring temperatures. However, for this to be done efficiently, ground handling agents will need to open up the package or content to monitor conditions that possibly expose the vaccines to extreme temperatures. Furthermore, this system allows inspection at the destination, and at that stage, it may become too late for personnel to take any corrective measure. Although financial constraints exist, fortunately in today’s artificial intelligence era, the emergence of sophisticated internet-of-things (IoTs), analytics, mobile and cloud technologies provide the basis for a comprehensive cold chain feedback mechanism. It allows the reporting of an ad-hoc and routine-base data collection, recording, checking, and analyses of the flow of vaccines from the manufacturing to healthcare facilities. Using a vaccine blockchain system and simulated machine learning technologies, Yong et al. presented a method of vaccine traceability effective in preventing vaccine record fraud and thus reducing supply risks. [32] Blockchain facilitates transparency and effectiveness in tracking, tracing and monitoring vaccine delivery. Using IoTs, more rigorous temperature monitoring in real time and forecasting can be achieved, although the processing of the generated data is questionable. Various aspects of cold chain generate experimental and numerical data that can train deep and machine learning models to predict temperature control, although according to Schroeder et al., a reliable method to detect a break in the cold chain is yet to be validated [33].

In addition, the use of digital temperature sensors such as thermocouples, resistant temperature detectors, or thermistors can provide accurate data. Purpose-built units or pharmaceutical grade units can also be used instead of household or dormitory-style refrigerators or freezers. The use of digital temperature tags has proven beneficial. For example, in India, vaccine manufacturers have begun airlifting vaccines in cold boxes with digital temperature tags to four major depots in four major states. These were then transported to specified facilities via airplanes or insulated vans after which they were stored in temperature-controlled facilities at the district level. Vaccines were held in ice-lined refrigerators in districts, then transported to distribution centres in cold boxes, which were then transported to vaccination sites in ice-packed vaccine carriers. Simultaneously, the COVID Vaccine Intelligence Network, a cloud-based digitalised platform for vaccine distribution management system, monitored the temperature of 29,000 cold-chain points in real-time [31].

In each vaccine vial, incorporation of a vaccine-vial monitor can provide a clear, visual guide to the vaccine's efficacy throughout the delivery or transport to the point of administration. In addition, the monitor is capable of warning personnel on whether or not the vaccines’ stability had been affected at any stage of distribution before reaching the end-users. This will prevent unnecessary wastage and facilitate vaccination outreach programmes in remote areas, where sophisticated monitoring technology is not practical [34].

### Improved vaccine formulation

As mRNA vaccine is considered new technology, there was a lack of guidance on the stability aspects of mRNA vaccines during the early phase of the vaccine development [35]. While it is common knowledge that there are a series of analytical methods used to determine the identity, purity, potency, safety, and stability of mRNA bioactive and mRNA–lipid/protein complex formulations, there is still a lack of relevant information on the monitoring of quality attributes and on the proposed storage conditions to ensure stability for mRNA vaccines.

Improvements in the stringent cold chain storage requirements can be made by improving vaccine formulation. One approach is to eliminate the cold chain altogether by making vaccines that can withstand more natural temperatures. Another method would be to stabilise vaccines through improved formulation, such as excipient innovation, protein engineering and lyophilisation if suitable. For instance, by making biologics such as an mRNA vaccine to withstand lyophilisation, its cold chain requirement might be lessened from deepfreeze to regular cold chain [12]. Few factors for consideration are selection of excipients (for example, stabilisers and/or the inclusion of preservatives), formulation milieu (for instance, pH and tonicifying agents), and manufacturing processes (for example, liquid to lyophilised dosage forms or powdered form) which can all be made without compromising vaccine potency. The improvised formulation may not require freezing conditions for its long-term storage. Improved understanding of the physicochemical processes underlying virus potency loss, combined with rational approaches to minimising their occurrence, would be highly beneficial in directing improved vaccine shelf life, which might eventually result in the abolition of 'cold chain' requirements.

A sustained interest in making vaccines safe at room temperature has resulted in several promising technologies—using specific polymers or sugars and optimising the composition of vaccines, certain vaccines can be rendered insensitive to freezing and/or stable for months at room temperatures [36]. In addition, transforming vaccines from liquid (or frozen liquid or frozen suspension) to dry powder for reconstitution at the point of administration can improve vaccine stability. Powder engineering technology such as shelf freeze-drying is widely used to transform small molecule pharmaceuticals, such as vaccines, and other biologics into dry powders while preserving consistency and sterility, which can then be stored for months, if not years, at room temperature [36]. This improved formulation of virus-based COVID-19 vaccines would result in improved stability profiles (such as shelf-life extension and conversion from freezer to refrigerator storage). Furthermore, the number of publications addressing mechanistic and systemic approaches to formulating and stabilising live attenuated virus and viral vector-based vaccine products is limited, indicating a critical area for future study. Another example is in the modification of its packaging; for example, for the oral cholera vaccine (Euvichol®), packaging them in plastic tubes rather than glass vials, significantly simplified the manufacturing method, reduced the unit cost, storage, and administration and contributed significantly to the WHO stockpile increase [37]. This had improved logistical obstacles, improved patient access and vaccine coverage. Similar innovations could also be emulated for COVID vaccines.

### Limitation of review

The lack of primary data on vaccine wastage or impact of a breach in the cold chain, for instance, has presented limitations in the reporting of the review. Waste handling post-vaccination is another major challenge which has not been addressed in this review. Disposal of primary packaging (vials and syringes), secondary packaging (cartons), and tertiary packaging (corrugating boards and cushioning materials) are valid concerns. Indeed, matters pertaining to the disposal of vaccine packaging material, and the disposal of personal protective equipment kits are of prime concern. A comprehensive set of guidelines for waste disposal has not been established, as yet.

## Conclusion

Vaccines are to be administered to all population in a timely manner. Establishing a secure cold chain management of global vaccine chain supply is critical. As a result, innovative technologies and techniques are needed to simplify vaccine distribution, by minimising the need for a cold chain, reducing packaging footprint, streamlining administration, and reducing waste. Potential practices include using renewable energy during production, storage, transportation, and waste treatment processes. In addition, equitable vaccine access can be promoted by using better packaging designs, utilising the Internet of Things and big data analytics for monitoring of logistics, as well as, managing real-time databases and coordination platforms to track vaccine deliveries and outreach to the relevant public vaccination programmes. Finally, COVID-19 vaccine storage, dosing, and scheduling could potentially vary over time, and there is, therefore, a need to continually educate and update healthcare providers, especially those who are involved in the vaccination programmes.

## Supplementary Information

**Additional file 1: Appendix S1.** Preferred Reporting Items for Systematic reviews and Meta-Analyses extension for Scoping Reviews (PRISMA-ScR) Checklist.

**Additional file 2: Appendix S2.** Full Search strategy – “Cold chain logistics for Covid-19 vaccines”.

**Additional file 3: Appendix S3.** Data charting form –Findings of the reviewed sources.

## Data Availability

All relevant data are within the manuscript.

## Abbreviations

PRISMA–ScR: Preferred Reporting Items for Systematic reviews and Meta-Analyses extension for Scoping Reviews
COVID-19: Coronavirus disease
SARS-CoV-2: Severe Acute Respiratory Syndrome Coronavirus 2
WHO: World Health Organisation
JKJAV: Special Committee for Ensuring Access to COVID-19 Vaccine Supply
NPRA: National Pharmaceutical Regulatory Agency
DEMATEL: Decision-making-trial-and-evaluation-laboratory
USA: United States of America
BCG: Bacillus Calmette–Guérin
MeSH: Medical Subject Headings
