# Supplementary material for: Management of COVID-19 vaccines cold chain logistics: a scoping review
Source: J Pharm Policy Pract. 2022 Mar 2;15:16. doi: 10.1186/s40545-022-00411-5 (PMC8889047; doi:10.1186/s40545-022-00411-5)
Supplement: Supplementary file 2 — Additional file 2: Appendix S2. Full Search strategy – “Cold chain logistics for Covid-19 vaccines”. [file 40545_2022_411_MOESM2_ESM.docx]

Additional File 2. Full Search strategy – “Cold chain logistics for Covid-19 vaccines”

Search filter – PUBMED (April 2020 – 1 July 2021)

| **Search** | **Query** |
| --- | --- |
| **#1** | (("COVID-19"[Mesh] OR "SARS-CoV-2"[Mesh] OR "COVID-19 Vaccines"[Mesh] OR "COVID-19 Serological Testing"[Mesh] OR "COVID-19 Nucleic Acid Testing"[Mesh] OR "SARS-CoV-2 variants" [Supplementary Concept] OR "COVID-19 drug treatment" [Supplementary Concept] OR "COVID-19 serotherapy" [Supplementary Concept] OR "2019-nCoV" OR "2019nCoV" OR "cov 2" OR "Covid-19" OR "sars coronavirus 2" OR "sars cov 2" OR "SARS-CoV-2" OR "severe acute respiratory syndrome coronavirus 2" OR “coronavirus 2” OR “COVID 19” OR “COVID-19” OR “2019 ncov” OR “2019nCoV” OR “corona virus disease 2019” OR “cov2” OR “COVID-19” OR “COVID19” OR  “nCov 2019” OR “nCoV” OR “new corona virus” OR “new coronaviruses” OR “novel corona virus” OR “novel coronaviruses” OR “SARS Coronavirus 2” OR “SARS2” OR “SARS-COV-2” OR “Severe Acute Respiratory Syndrome Coronavirus 2”) AND (2020/4/1:3000/12/31[PDAT])) |
| **#2** | ((“Cold Chain” [Mesh] OR “Chain, Cold” OR “Chains” [Mesh], “Cold OR Cold Chains” [Mesh] OR Vaccine vial monitor OR Thermostab* (thermostable, thermostability, thermostabilization) OR supply AND distribution* |
| **#3** | (Vaccination[MeSH Terms]) OR "Immunisation programme"[Title/Abstract]) OR Vaccine*[MeSH Terms]) OR "Immunization, immunisation"[Title/Abstract]) OR innoculation[MeSH Terms]) OR "Vaccine"[Title/Abstract] OR (Viral vaccine*) OR Covid-19 vaccine* |
| **#4** | 1 AND 2 AND 3 |
