# Supplementary material for: Management of COVID-19 vaccines cold chain logistics: a scoping review
Source: J Pharm Policy Pract. 2022 Mar 2;15:16. doi: 10.1186/s40545-022-00411-5 (PMC8889047; doi:10.1186/s40545-022-00411-5)
Supplement: Supplementary file 3 — Additional file 3: Appendix S3. Data charting form –Findings of the reviewed sources. [file 40545_2022_411_MOESM3_ESM.docx]

| Authors | Origin | Purpose | Type of source | Research design | Process Target | Conceptual/ theoretical framework | Framework proposed | Major themes |
| --- | --- | --- | --- | --- | --- | --- | --- | --- |
|  |  |  |  |  |  |  |  |  |

Appendix 3: Data charting form –Findings of the reviewed sources
